# Analgesic effect of the ultrasound-guided subcostal approach to transmuscular quadratus lumborum block in patients undergoing laparoscopic nephrectomy: a randomized controlled trial

**DOI:** 10.1186/s12871-019-0825-4

**Published:** 2019-08-14

**Authors:** Manhua Zhu, Yong Qi, Huijuan He, Jinfeng Lou, Qingqing Pei, Yuliu Mei

**Affiliations:** 1 Department of Anesthesiology, Ningbo Medical Center Lihuili Eastern Hospital, Taipei Medical University Ningbo Medical Center, No.1111 jiangnan Road, Ningbo, Zhejiang, 315040 China; 2 Department of Anesthesiology, Ningbo Beilun People’s Hospital, Ningbo, Zhejiang, China, No.1288 lushan east Road, Ningbo, 315800 China

**Keywords:** Quadratus lumborum block, Laparoscopic nephrectomy, Pain, Postoperative

## Abstract

**Background:**

Quadratus lumborum block (QLB) is an effective analgesia that lowers opioid consumption after lower abdominal and hip surgeries. The subcostal approach to transmuscular QLB is a novel technique that can provide postoperative analgesia by blocking more dermatomes. The aim of this study is to evaluate the efficacy and viability of subcostal approach to QLB after laparoscopic nephrectomy.

**Methods:**

Sixty patients who underwent laparoscopic nephrectomy were randomly divided into the subcostal approach to QLB group (QLB group, *n* = 30) and the control group (C group, *n* = 30). All patients underwent ultrasound-guided subcostal approach to QLB in an ipsilateral parasagittal oblique plane at the L1–L2 level. The QLB group received 0.4 cc/kg of 0.3% ropivacaine, and the C group received 0.4 cc/kg of 0.9% saline. Postoperatively, a patient-controlled intravenous analgesic pump with sufentanil was attached to all the patients. The primary outcome was sufentanil consumption within the first 24 h after surgery. The secondary outcomes included the Ramsey sedation scale (RSS) scores and Bruggemann comfort scale (BCS) scores 6 h (T1), 12 h (T2), and 24 h (T3) after surgery, intraoperative remifentanil consumption, number of patients requiring rescue analgesia, time to recovery of intestinal function, mobilization time after surgery, and presence of side effects.

**Results:**

Sufentanil consumption within the first 24 h after surgery was significantly lower in the QLB group than in the C group (mean [standard deviation]: 34.1 [9.9] μg vs 42.1 [11.6] μg, *P* = .006). The RSS scores did not differ between the two groups, and the BCS scores of the QLB group at T1 and T2 time points was significantly higher than those of the C group(*P*<0.05). The consumption of remifentanil intraoperatively and the number of patients requiring rescue analgesia were significantly lower in the QLB group (*P*<0.05). Time to recovery of intestinal function and mobilization time after surgery were significantly earlier in the QLB group (*P*<0.05). The incidence of postoperative nausea and vomiting was significantly lower in the QLB group (*P*<0.05).

**Conclusions:**

The ultrasound-guided subcostal approach to QLB is an effective analgesic technique in patients undergoing laparoscopic nephrectomy as it reduces the consumption of sufentanil postoperatively.

**Trial registration:**

ChiCTR1800020296 0 (Prospective registered). Initial registration date was 22/12/2018.

## Background

The laparoscopic technique is more often used in nephrectomy than in open surgeries, and it has numerous advantages, such as smaller incision and rapid recovery. However, postoperative pain caused by pneumoperitoneum and surgical manipulations of the kidneys should not be underestimated. Postoperative pain and stress response will aggravate patients’ disease, increase the incidence of complications, and prolong postoperative recovery. As an important element of multimodal analgesia, regional blocks can reduce the dosage of opioids, minimize side effects, and enhance the quality of recovery after surgery [1].

Quadratus lumborum block (QLB) is an emerging truncal block technique, [2] which includes injecting local anesthetic (LA) into the thoracolumbar fascia (TLF) surrounding the quadratus lumborum (QL) muscle. The analgesic effect is produced by the LA spreading along the TLF into the thoracic paravertebral space and transversalis fascia. QLB is an effective analgesic method for patients undergoing abdominal and hip surgeries [3–6]. Based on the different injection sites, there are four types of QLB, namely, lateral QLB, posterior QLB, transmuscular QLB, and intramuscular QLB. Transmuscular QLB, which is also called QLB3, includes the injection of LA between the QL muscle and the psoas major (PM) muscle. QLB3 can be implemented at the L4 and L2 levels using the subcostal approach [7]. Hesham et al. [8] have reported that the use of the subcostal approach to QLB3 can provide appropriate sensory blockade for open urological surgeries. However, no randomized controlled trials have assessed the application of the subcostal approach to QLB in patients undergoing laparoscopic nephrectomy. Thus, the current study aimed to evaluate the postoperative analgesic efficacy and the viability of the subcostal approach to QLB in patients undergoing laparoscopic nephrectomy with postoperative opioid consumption and self-reported sedation and comfort scores.

## Methods

The study protocol was approved by the Ethics Committee of Ningbo Medical Center, Lihuili Eastern Hospital, China (DYLL2018073). This study has been registered at the Chinese Clinical Trial Registry (ChiCTR1800020296). Sixty patients undergoing laparoscopic nephrectomy under general anesthesia were enrolled between January 2019 and March 2019 in Ningbo Medical Center Lihuili Eastern Hospital. The inclusion criteria were as follows: patients with an American Society of Anesthesiologists (ASA) physical status I–III and those aged between 35 and 65 years. Meanwhile, the exclusion criteria included patients with serious cardio-cerebral vascular diseases, allergies to LAs, infection at the puncture site, body mass index (BMI)>35 kg/m^2^, history of mental illness, language communication disorder and patients who did not consent to the procedure.

**Fig. 1:**
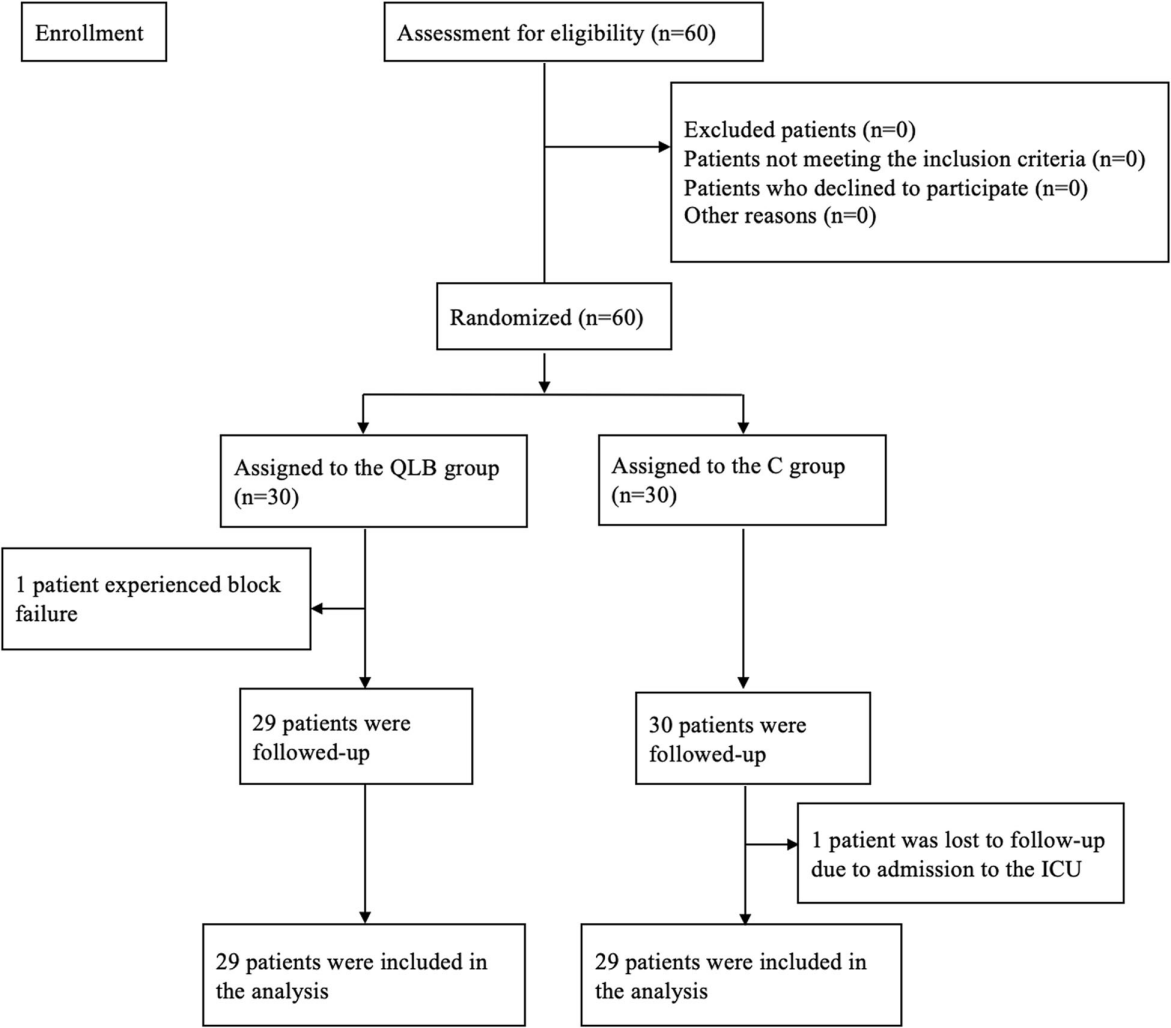
Flowchart of this study

After obtaining informed consent, the patients were randomly allocated into two groups using a computer-generated random table (GraphPad Software, Inc., La Jolla, CA, USA): the subcostal approach to QLB group (QLB group) and the control group (C group) (Fig. 1).

Standard monitoring was performed after the patients were transported to the operating room. The patient’s heart rate, electrocardiography results, percutaneous oxygen saturation, invasive radial arterial pressure, end-tidal carbon dioxide, and bispectral index (BIS) were assessed. Next, a peripheral vein access and right internal jugular vein access were established, and infusion of Ringer’s lactate solution was initiated intravenously.

**Fig. 2:**
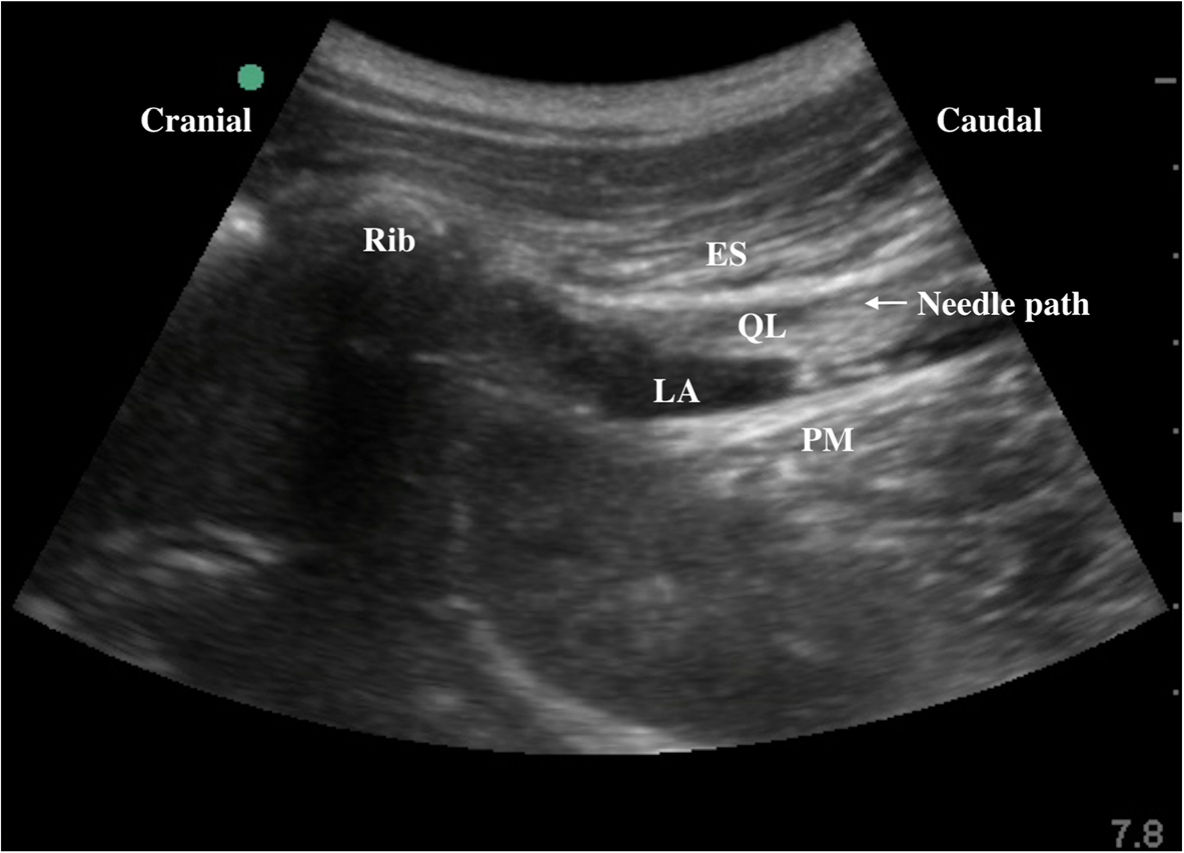
Ultrasound image of the subcostal approach to transmuscular QLB. LA spread betweem QL and PM muscle. *LA* local anesthetic, *QL* quadratus lumborum muscle, *ES* erector spinae muscle, arrow shows the needle path

Prior to induction of general anesthesia, 0.02 mg/kg of midazolam was administered to the patients intravenously, and the patients were placed in the lateral position. Following disinfecting of the surgical area, a convex probe (2–5 HZ, Edge, Sonosite, Seattle, the USA) was positioned below the 12th rib in a parasagittal oblique plane at the L1–L2 level, which is approximately 4 cm from the posterior midline. The 12th rib, erector spinae (ES) muscle, QL muscle, and PM muscle were identified, and a 22-gauge, 80-mm ultrasound visible needle (Kindly, Shanghai, China) was directed to the anterior part of the QL. Then, the needle tip was located between the QL and PM using the in-plane technique. After confirming the site via hydrodissection, 0.4 cc/kg of 0.3% ropivacaine (Naropin, AstraZeneca AB Company, Sodertalje, Sweden) was injected between the QL and PM muscles in the QLB group. In addition, 0.4 cc/kg of 0.9% saline was injected at the same site in the C group (Fig. 2). An experienced anesthesiologist performed all blocks. The patients, anesthesiologists, surgeons, and nurses were all blinded to the study.

After performing the block, all patients received general anesthesia. Anesthesia was induced with intravenous propofol 2–2.5 mg/kg, sufentanil 0.4 μg/kg, and rocuronium 0.8 mg/kg. A tracheal intubation was performed and mechanical ventilation initiated. A maintainace dose of propofol 0.1–0.15 mg/kg/min and remifentanil 0.1–0.3 μg/kg/min to maintain a BIS of 40 to 60 was administered. 15 min to the end of the surgery, sufentanil 0.15 μg/kg and parecoxib 40 mg were administered intravenously for postoperative pain control. Postoperatively, a patient-controlled intravenous analgesic (PCIA) pump with 1 μg/mL of sufentanil was attached to all the patients. The device was adjusted to deliver 2 mL of intravenous bolus on demand, with no background infusion and a 15-min lockout interval. Postoperative pain was assessed with a 10-point visual analogue scale (VAS) (0, no pain, 10, worst imaginable pain). Intravenous dezocine 5 mg was used for rescue analgesia when the VAS pain score at rest was greater than 4.

The primary outcome measure was the amount of sufentanil consumption within the first 24 h after surgery.

The secondary outcome measures were the Ramsey sedation scale (RSS) and Bruggemann comfort scale (BCS) scores 6 h (T1), 12 h (T2), 24 h (T3) after surgery, heart rate (HR) and median arterial pressure (MAP) before anesthesia (t0), 5 min after skin incision (t1) and the end of surgery (t2), intraoperative remifentanil consumption, number of patients requiring rescue analgesia, time to recovery of intestinal function (time from recovery to the first flatus), mobilization time after surgery, and presence of side effects (postoperative nausea and vomiting [PONV]), respiratory depression, femoral nerve block, LA systemic toxicity, and local hematoma). After performing the block, the dermatomes of the sensory block were assessed 30 min later using pinprick in both groups.

The RSS (1, anxious, agitated and restless; 2, cooperative, oriented and tranquil; 3, responsive to commands only; 4, brisk response to light glabellar tap or loud auditory stimulus; 5, Sluggish response to light glabellar tap or loud auditory stimulus; 6, no response) and the BCS (0, continuous pain; 1, painless without movement, sever pain while breathing deeply or coughing; 2, painless without movement, mild pain while breathing deeply or coughing; 3, painless when breathing deeply; 4, painless when coughing) were applied to patients by two nurses who were blinded to the group allocation. The RSS was used to measure the sedation level and the BCS was used to evaluate analgesic efficacy.

Our study was powered using a pilot study to detect the sufentanil consumptions within the first 24 h after surgery between the QLB group and the C group. Pilot study that included 12 patients showed a mean sufentanil consumptions of 28.4 μg with standard deviation of 4.74 24 h after surgery in the QLB group and 33.3 μg with standard deviation of 5.74 in the C group. Based on these data, we determined that a total of 42 patients were required (21 in each group), which was calculated by IBM SPSS Sample Power v3.0 (IBM Corp., Armonk, New York, the USA) at a power of 0.8 with 0.05 alpha. Thus, we recruited 30 patients for each group to account for the possibility of missing data or dropouts.

The Statistical Package for the Social Sciences software version 24.0 (IBM Corp., Armonk, New York, USA) was used in all statistical analyses.

Data were collected and entered into the computer as numerical or categorical data (IBM SPSS Statistics for Windows, version 24.0, IBM Corp., Armonk, New York, the USA). Complete descriptive statistics were recorded for each variable, including mean, standard deviation, median, and interquartile range. The Kolmogorov–Smirnov test was used to determine whether the variables were normally distributed. The independent *t*-test or Mann–Whitney U test was used for the intergroup comparisons accordingly. The chi-square (*χ*^2^) test or Fisher exact test was used to compare qualitative variables. The rank-sum test was used to compare skewed distribution variables. For all comparisons, a *P* value < 0.05 was considered statistically significant, and the differences were then identified.

## Results

A total of 60 patients were included in our study. One patient in the QLB group was excluded due to block failure, and one patient in the C group was lost to follow-up; 29 patients in each group completed the study The Consolidated Standards of Reporting Trials (CONSORT) diagram is shown in Fig. 1. The two groups did not differ in terms of age, gender, BMI, ASA physical status, and operative characteristics (operative time and type of surgery) (Table 1).

**Table 1:** Demographic and operative characteristics

		QLB group (*n* = 29)	C group (*n* = 29)	*P*
Age (years)		49.3 ± 10.1	54.2 ± 8.3	0.153
Sex ratio [case (%)]	Male	13 (44.8%)	15 (51.7%)	0.599
	Female	16 (55.2%)	14 (48.3%)	
Body mass index (kg/m^2^)		24.0 ± 2.4	23.4 ± 3.1	0.554
ASA [case (%)]	I	13 (44.8%)	14 (48.3%)	0.689
	II	12 (41.4%)	13 (44.8%)	
	III	4 (13.8%)	2 (6.9%)	
Operative time (min)		63.1 ± 13.3	64.7 ± 15.5	0.764
Type of surgery [case (%)]	radical nephrectomy	12 (41.4%)	11 (37.9%)	0.788
	partial nephrectomy	17 (58.6%)	18 (62.1%)	

Data are presented as mean ± standard deviation or number (%). QLB group, patients who received a combination of general anesthesia with quadratus lumborum block; C group, patients who received general anesthesia.

ASA = American Society of Anesthesiologists.

### Primary outcome

**Fig. 3:**
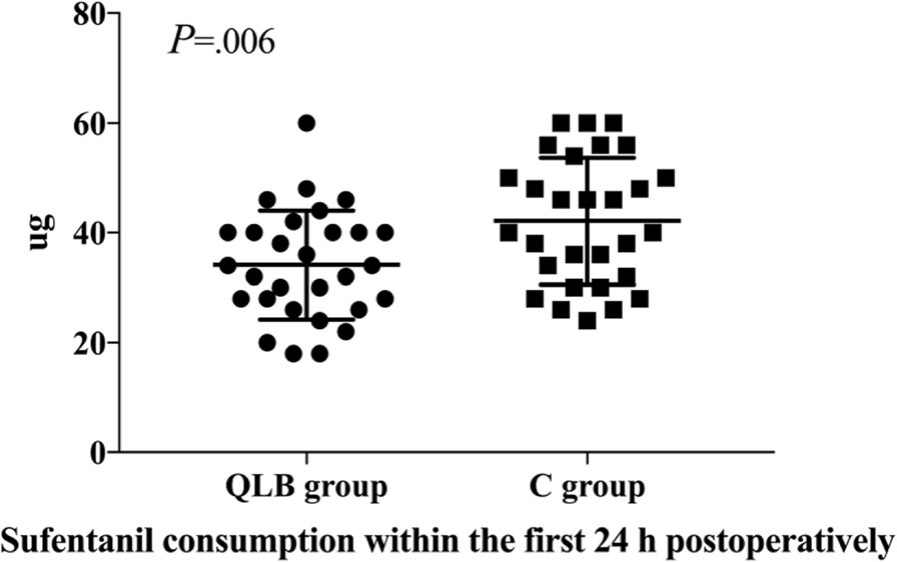
Sufentanil consumption within the first 24 h postoperatively for the QLB and control groups. Data are presented as individual values and mean ± standard deviations. QLB group (closed circles, *n* = 29), patients who received a combination of general anesthesia with quadratus lumborum block; C group (closed squares, *n* = 29), patients who received general anesthesia. (*P* = 0.006)

Patients in the QLB group had lower sufentanil consumption within the first 24 h postoperatively than the C group (mean [standard deviation]: 34.1 [9.9] μg vs 42.1 [11.6] μg, t = 2.829, *P* = 0.006) (Fig. 3).

### Secondary outcomes

**Fig. 4:**
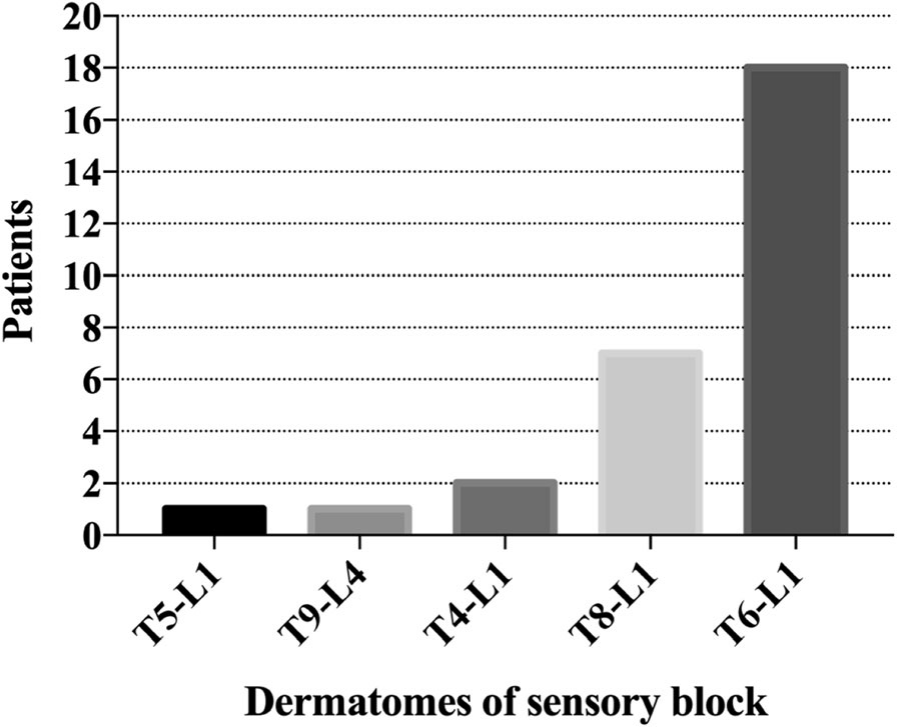
Dermatomes of the sensory block in the patient who received QLB. Frequency of sensory segments blockade to sharp touch at 30 min following QLB

The Ramsey sedation scale (RSS) scores did not differ at any timepoint between the two groups, and the Bruggemann comfort scale (BCS) scores were higher in the QLB group than in the C group at all time points. Significant differences were observed at T1 and T2 time points (Table 2). HR and MAP were significantly lower at t1 in the QLB group than in the C group, and there were no significant differences at t0 and t2 between the two groups; only HR and MAP were significantly higher at t1 than t0 in the C group (Table 3). The intraoperative consumption of remifentanil was significantly lower in the QLB group than in the C group. The number of patients requiring rescue analgesia was significantly lower in the QLB group than in the C group, and the time to recovery of intestinal function and mobilization time after surgery were significantly earlier in the QLB group than in the C group (Table 4). The incidence of PONV was significantly lower in the QLB group than in the C group (Table 4). Only one patient in the QLB group presented with femoral nerve block that manifested as lower limb weakness, the duration of which was approximately 10 h. Neither group presented with respiratory depression, LA systemic toxicity, or local hematoma. Thirty minutes after the application of QLB, the dermatomes of the sensory block in the QLB group were maintained at T4–L2, and the main blocking area was at T6–L1 (Fig. 4).

**Table 2:** Sedation scale and comfort scale score [Score, M (IQM)]

	QLB group	C group	*P*
Ramsay sedation scale			
T1	2.0 (2,3)	3.0 (2,3)	0.472
T2	2.0 (2,2)	2.0 (2,3)	0.671
T3	2.0 (2,2)	2.0 (2,2)	0.671
Bruggemann comfort scale			
T1	3.0 (2,3)	2.0 (1,3)	0.017
T2	3.0 (2,3)	2.0 (2,3)	0.038
T3	3.0 (3,4)	3.0 (2,3)	0.293

Data are presented as median (IQR). IQR, interquartile range. QLB group, patients who received a combination of general anesthesia with quadratus lumborum block; C group, patients who received general anesthesia.

**Table 3:** intraoperative HR and MAP (mean ± standard deviation)

	group	t0	t1	t2
HR (bpm)	QLB	78.65 (11.6)	83.24 (11.7) ^a^	82.34 (10.6)
	C	79.86 (13.3)	97.14 (10.2) ^b^	78.28 (12.6)
MAP (mmHg)	QLB	100.97 (8.7)	104.52 (8.9) ^a^	101.5 (8.6)
	C	100.55 (9.1)	113.45 (12.0) ^b^	102.0 (10.3)

Data are presented as mean ± standard deviation. QLB group, patients who received a combination of general anesthesia with quadratus lumborum block; C group, patients who received general anesthesia.

Co mpared with C group, ^a^*P* < 0.05; Compared withT0, ^b^*P* < 0.05.

**Table 4:** Consumption of remifentanil intraoperatively, postoperative conditions, and PONV

	QLB group	C group	*P*
Consumption of remifentanil intraoperatively (μg)	357.3 ± 66.7	445.3 ± 72.6	0.002
Number of patients requiring rescue analgesia (%)	6 (20.7%)	18 (62.1%)	0.001
Time to recovery of intestinal function (h)	54.7 ± 6.6	62.6 ± 6.2	0.002
mobilization time (h)	25.4 ± 4.1	29.9 ± 4.3	0.006
PONV (%)	8 (27.6%)	16 (55.2%)	0.033

Data are presented as mean ± standard deviation or median (%) number of patients. QLB group, patients who received a combination of general anesthesia with quadratus lumborum block; C group, patients who received general anesthesia.

PONV = postoperative nausea and vomiting.

## Discussion

Although laparoscopic nephrectomy has the advantages of small incision and rapid recovery, early postoperative pain including incision pain, visceral pain and tube-related stress can still not be underestimated. It can cause a series of physiological and psychological reactions [9], such as increase the oxygen consumption, inhibition of respiration and reduce life quality of patients. Therefore, it’s very important to improve postoperative analgesia, raise patient comfort and reduce the complication rates [10].

In recent years, with the maturity of ultrasound-guided nerve block techniques, QLB is widely used in clinic to provide postoperative analgesia for abdominal and hip surgeries [3–6]. There are several approaches to QLB with different block planes, and the subcostal approach was first described by Elsharkawy [11] in 2016. LA was injected into the space between the QL muscle and PM muscles and it spreads to the paravertebral space through the arcuate ligaments in a cephalad direction. The anatomical basis of the spread pattern is that both the PM and QL muscles insert into the thoracic cavity in a funnel-shaped manner [12].

The subcostal approach to QLB was reported to achieves a wider block range between T6–T7 and L1–L2; and it can provide effective postoperative analgesia for patients undergoing lower abdominal surgery [13] and hip arthroplasty [14]. To the best of our knowledge, this randomized prospective study is the first to investigate the use of the subcostal approach to QLB in laparoscopic nephrectomy.

The lateral abdominal wall is the common surgical incision site in laparoscopic nephrectomy, [15] which is innervated by the anterior branches of T8–L1 spinal nerves. Transection of the skin, muscle, and peripheral nerves can cause severe postoperative pain. At present, most of the analgesic methods used in clinical settings have some disadvantages. Opioids can cause respiratory depression, nausea, and vomiting, and epidural analgesia can cause nerve injury and epidural hematoma [16]. The analgesic effect of NSAIDS is limited, and the incidence of nephrotoxicity is high [17]. As an important element of the multimodal analgesia program, regional block is a type of analgesia with high safety and less adverse effects [18]. We chose the subcostal approach to QLB in the study because there are several advantages of this method. (1) It can provide a higher sensory block plane due to higher needle insertion, and we can observe the extent of LA spread in the parasagittal oblique plane. (2) it is relatively safe, because the tip of the needle was placed between the QL and PM muscle, and this can prevent intraperitoneal injection and kidney injury.

Our results showed that sufentanil consumption on postoperative day 1 and the number of patients requiring rescue analgesia were significantly lower in the QLB group than in the C group, which was in accordance with the findings of the study of Blanco et al., [4] who first described the QLB, and that study also found that QLB significantly reduces the consumption of morphine after cesarean delivery. Baidya et al. [19] have reported that QLB is associated with minimal requirements for rescue analgesics, and offers adequate postoperative analgesia in children undergoing pyeloplasty. LA can spread into the paravertebral space and the thoracolumbar plane in QLB. Tesarz et al. [20] have revealed that the thoracolumbar fascia contains high density sympathetic fibers and pain receptors, and QLB can alleviate both somatic and visceral pain partially due to the blockade of these receptors [21]. Our study showed that the subcostal approach to QLB blocks the sensory nerves from T4–L1, and can provide adequate analgesia after laparoscopic nephrectomy.

The RSS scores of the two groups in our study did not differ, whereas the BCS scores were higher in the QLB group than in the C group. The results further showed that the analgesic effect of the subcostal approach to QLB was sufficient. In addition, the fluctuations of hemodynamic parameters during the operation were smaller in QLB group than C group and the intraoperative consumption of remifentanil in the QLB group was lower than that in the C group, and the result was in accordance with the findings of the study by Naglaa et al., [22] who reported that QLB is associated with less opioid consumption. The results of our study indicated that the subcostal approach to QLB combined with general anesthesia can keep vital signs relative stable and reduce the required dose of opioids intraoperatively. It can be explained by the assumption that LA spreads to the paravertebral space and the sympathetic trunk to produce an analgesic effect and inhibit the stress response of surgery.

Moreover, in the present study, the time to recovery of intestinal function and mobilization time of the QLB group were significantly earlier than those of the C group, which is consistent with the results reported by Zhu et al. [23] The shorter time to the first flatus in the QLB group can be attributed to less consumption of sufentanil postoperatively. Bowel dysfunction is a side effect induced by opioids, which include constipation and slow peristalsis in the intestine. The QLB group received adequate analgesia and had early ambulation with mild postoperative pain. Early food taking and mobilization are two important components of enhanced recovery after surgery [1]. Thus, we speculated that the subcostal approach to QLB can promote the rehabilitation of patients after laparoscopic nephrectomy.

In the current study, the incidence of PONV was lower in the QLB group than in the C group, and it may be associated with less consumption of opioids. Consistent with the case reported by Wikner et al. [24] and Hockett et al., [25] one patient in the QLB group presented with femoral nerve block partly due to the spread of LA to the lumbar paravertebral space and blockade at part of the lumbar plexus. If we injected LA between the middle layer of the thoracolumbar fascia (TLF) and the QL muscle, we suspect that the incidence of femoral nerve block would decline because TLF restricts the spread of LA. However, further studies must be conducted to confirm this result.

### Limitations

The present study has some limitations. First, the subcostal approach to QLB was not used in patients with BMI > 35 kg/m^2^. Thus, we did not identify the efficiency of the subcostal approach to QLB in obese patients. Second, the small sample size may limit the identification of the adverse effects of the subcostal approach to QLB. Third, although none of the patients in the subcostal approach to QLB group presented with LA systemic toxicity, further studies must be conducted to validate optimal LA concentrations and volumes.

## Conclusions

The ultrasound-guided subcostal approach to QLB in patients undergoing laparoscopic nephrectomy reduced the postoperative consumption of sufentanil and provided a more effective postoperative analgesia; as a result, a lower number of patients required rescue analgesia. We conclude that subcostal approach to QLB is an effective analgesic technique in patients undergoing laparoscopic nephrectomy.

## Data Availability

The datasets used during the current study are available from the corresponding author on reasonable request.

## Abbreviations

ASA: America Society of Anesthesiologist
BCS: Bruggemann comfort scale
BIS: Bispectral index
BMI: Body mass index
CONSORT: Consolidated Standards of Reporting Trials
ES: Erector spinae
HR: Heart rate
IQR: Interquartile range
LA: Local anesthetic
MAP: Median arterial pressure
NSAIDS: Nonsteroidal antiinflammatory drugs
PM: Psoas major
PONV: Postoperative nausea and vomiting
QLB: Quadratus lumborum block
RSS: Ramsey sedation scale
TLF: Thoracolumbar fascia
VAS: Visual analogue scale
